# Mitral Kissing Vegetation and Acquired Aortic Valve Stenosis Secondary to Infectious Endocarditis in a Goat with Suppurative Mastitis

**DOI:** 10.3390/vetsci5030064

**Published:** 2018-07-10

**Authors:** Antonio Watson, Vade Sookram, Marc Driscoll, Michael Morris, Rod Suepaul, Jordi López-Alvarez, Ignacio Corradini

**Affiliations:** 1 Department of Clinical Veterinary Sciences, School of Veterinary Medicine, The University of the West Indies, EWMSC, Mt. Hope, Trinidad and Tobago; antonio_watson@hotmail.com (A.W.); vnsvade47@gmail.com (V.S.); madriscoll.dvm@gmail.com (M.D.); vetondenet@hotmail.com (M.M.); 2 School of Veterinary Medicine, The University of the West Indies, EWMSC, Mt. Hope, Trinidad and Tobago; Rod.Suepaul@sta.uwi.edu; 3 Fundació Hospital Clínic Veterinari, Universitat Autònoma de Barcelona, Cerdanyola del Vallès, 08290 Barcelona, Spain; cardiologia.hcv@uab.cat; 4 Hospital Clínico Veterinario, Dpto. de Medicina y Cirugía Animal, Facultad de Veterinaria, Universidad Cardenal Herrera-CEU, CEU Universities, Alfara de Patriarca, 46115 Valencia, Spain

**Keywords:** caprine, echocardiography, left ventricular outflow tract, valvular stenosis, secondary mitral infection, mitral kissing vegetation, aortic stenosis, mastitis, infective endocarditis

## Abstract

A six-year-old female goat was presented to the veterinary teaching hospital of the University of the West Indies with a history of progressive hind-limb paresis lasting two weeks. The doe developed a grade 6/6 holosystolic murmur during hospitalisation. Echocardiography revealed vegetative growths attached to cusps of the mitral and aortic valves. There was an accelerated aortic flow at 2.9 m/s and aortic insufficiency. The aortic vegetation was prolapsing into the left ventricle during diastole, causing it to contact the septal mitral valve leaflet. A diagnosis of mitral and aortic vegetative endocarditis, with a mitral kissing vegetation and mild aortic stenosis, was reached. The patient was placed on broad-spectrum antimicrobials. A short-term follow-up showed no resolution of clinical signs, and the animal eventually died. Post-mortem examination showed severe vegetative, fibrino-necrotic, aortic and mitral valve lesions. The goat also had a severe fibrino-suppurative mastitis. Histopathology confirmed the lesions to be vegetative endocarditis.

## 1. Introduction

Infective endocarditis remains a highly morbid disease that affects the endocardium. Infective endocarditis has been reported in ruminants including bovine, sheep, and camelids. However, infectious endocarditis has been rarely described in goats, with only a few documented cases in scientific literature [1]. In large animals, endocarditis commonly occurs secondarily to disseminated sepsis originating from infection in a distant site. It has been documented that animals with conditions, such as chronic active infections, including foot abscesses or rumenitis, can develop bacteraemia, which can then be a predisposing factor for endocarditis [2]. However, this has not been commonly reported in goats. Both mitral kissing vegetation and acquired valvular obstruction, although rare, are possible complications of infective endocarditis in human beings. However, these complications have been scarcely reported in veterinary medicine. A mitral kissing vegetation results from a large aortic vegetation prolapsing into the left ventricular outflow tract while making contact with the ventricular aspect of the anterior mitral leaflet, thus causing secondary infection [3,4,5]. Mitral kissing vegetation has not been previously described in animal species. Acquired valvular obstruction, however, most commonly occurs secondary to calcific degeneration in human beings and enzootic calcinosis in animals [6,7,8,9]. Acquired valvular stenosis due to infective endocarditis is uncommon in animals and has not been described in goats.

This case report describes a goat with weakness, a mammary abscess, and infectious endocarditis with large vegetative lesions on the aortic valve, causing mild aortic stenosis and a “kissing vegetation” of the mitral valve.

## 2. Case Description

A six-year-old dairy Saanen doe was presented to the veterinary teaching hospital of the University of the West Indies in sternal recumbency for the previous two days and with a history of left hind limb lameness over the previous two weeks. The animal weighed 55.5 kg and had a body condition score of 2/5, with a high average milk production of 5 L of milk per day. She was estimated to be three months pregnant based on a foetal crown–rump length measurement by a trans-abdominal ultrasonographic examination (Easi-Scan 2 Ultrasound scanner^TM^, BCF Technology Ltd., Bellshill, Scotland). The doe was recumbent but bright, alert, and responsive. The respiratory rate was markedly elevated at 84 breaths per minute (BrPM) (Reference range (RR): 15 to 30 BrPM) [1]. The heart rate was mildly elevated at 92 beats per minute (BPM) (RR: 70 to 90 BPM) [1], and the rectal temperature was moderately increased at 40.2 °C (RR: 39.0 °C ± 0.5 °C) [1]. The doe had two strong rumen contractions in 2 min (RR: 1 to 1.5 in 2 min). There was an absence of pain and swelling in both halves of the udder, and the milk was normal in appearance. There was a partial thickness wound on the right teat, which was associated with healthy granulation tissue. The doe was unable to stand without assistance, but it made repeated attempts by rising on to its carpi. Upon orthopaedic examination, the left fetlock was slightly swollen, with a decreased range of motion. A neurological examination revealed marked weakness in the hind limbs, with an intact panniculus reflex and weak myotactic reflexes of both the fore and hind limbs. Radiographic views of the affected left fetlock identified mineralisation around the joint. Views of the cervical, thoracic, and lumbar vertebrae demonstrated no abnormalities.

Blood was taken for both complete blood count and serum biochemistry, but as the animal was received outside of normal working hours, the results were delayed until the following day.

At the time of presentation, hypocalcaemia was suspected because the doe was a heavy milker, with weak pelvic limb reflexes and paresis. Emergency treatment was initiated, administering 60 millilitres of 23% calcium borogluconate (Cal-Plus®, Vetoquinol N-A. Inc., 2000, ch. Georges, Lavaltrie, QC, Canada) as a slow intravenous (IV) infusion, with simultaneous cardiac auscultation. 

On the following day, the eventual haematological results revealed a marked leukocytosis due to neutrophilia, lymphocytosis, and monocytosis. The plasma protein was increased at 83 g/L, and fibrinogen was within normal ranges. The serum calcium was slightly decreased (Table 1). Due to the clinical findings and haematological evidence of marked systemic inflammation (Table 1), the doe was started on broad-spectrum antibiotic therapy with penicillin and streptomycin (Combikel® 20/20, KELA N.V./St. Lenaartsewg 48 2320, Hoogstraten, Belgium) (30,000 IU/kg of penicillin once daily) delivered intramuscularly, and a non-steroidal anti-inflammatory drug, flunixin meglumine (Banamine®, Intervet International B.V., Boxmeer, The Netherlands), administered once daily at a dose of 2 mg/kg IV.

### 2.1. WBC, White Blood Cells

On Day 8, the doe stood for the first time with assistance for a period of one minute. Upon cardiac auscultation, a gallop sound was detected in the morning, but it was absent later in the day. A repeat complete blood count showed a leukocyte count within the reference range. However, the neutrophil count was still mildly elevated at 9.02 × 10^9^/L. Physical therapy was initiated, consisting of daily hoisting, within a harness in a float tank, with passive range of motion exercises. Progression over the following 12 days was fair: The animal remained bright and alert, with a normal respiratory rate and temperature, but with a mild tachycardia. However, hind limb paresis, muscular strength, and reflexes did not change. On thoracic auscultation, there were intermittent inspiratory crackles over the left lung field. On the 14th day of hospitalisation, a gallop sound was again transiently auscultated in the morning. Heart sounds remained normal until the 20th day of hospitalisation, when a systolic, band-shaped, grade 5/6 murmur was auscultated with a maximal intensity in the left aortic (axilla) region. The heart rate was 108 BPM.

### 2.2. Echocardiographic Examination (Day 20)

**Figure 1:**
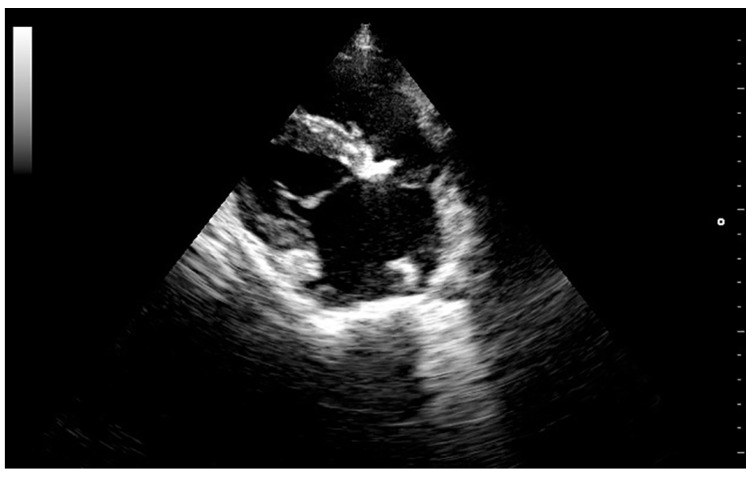
Right parasternal longitudinal 4-chamber view showing increased echogenicity and enlargement of the Mitral valve.

**Figure 2:**
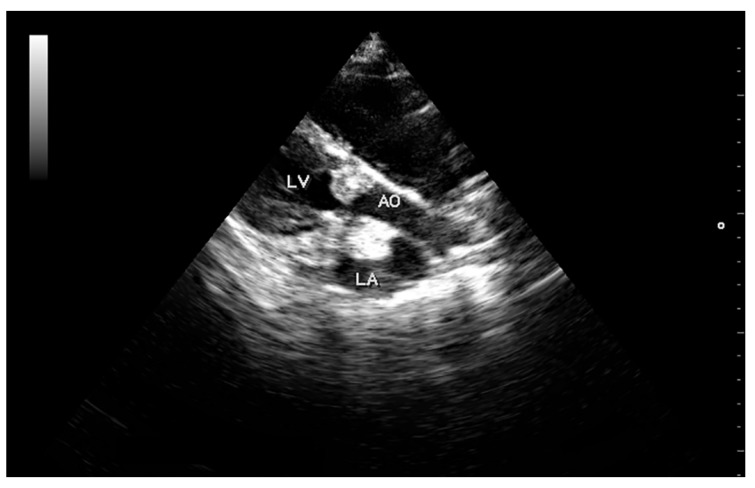
Right parasternal longitudinal view of the left ventricular outflow tract showing large vegetations over an aortic valve cusp and above the mitral valve protruding into the left atrium. Abbreviations: LV, left ventricle; AO, aorta; LA, left atrium.

High-resolution images in B and M-mode were obtained (Mylab 30TM, Esaote North America, Inc., Indianapolis, Indiana). The trans-thoracic echocardiogram showed a 1.5 cm vegetative growth on the mitral valve, which was protruding into the left atrium right above the septal cusp of the mitral valve. The mitral valve leaflets were hyperechogenic and thickened (Figure 1). In addition, one of the aortic valve leaflets (the left coronary cusp) was markedly thickened, presenting a 0.6 cm vegetative growth over the body and tip of the leaflet (Figure 2). The affected aortic valve leaflet had a markedly decreased range of motion during systole. However, this cusp was prolapsing into the left ventricle (LV) during diastole, causing it to “kiss” the septal mitral valve cusp (Video I: available in Supplemental Material, online). Severe mitral and tricuspid regurgitation were also observed from the left and right parasternal views, respectively. The location of the regurgitation jets, as well as the aortic flow, were verified using colour Doppler, until a flow signal with a maximum spectral representation of high velocities was obtained. The peak velocity of the aortic flow was 2.9 m/s (estimated peak pressure gradient was 31.3 mmHg). Due to the abnormal appearance of the aortic valve, the hypomotility of the affected cusp during systole, and the abnormal peak pressure gradient, mild acquired aortic stenosis was diagnosed.

Blood was collected aseptically for a bacteriological culture, but unfortunately, it yielded a negative result. Due to a worsening clinical picture, the antibiotic therapy was changed to ceftriaxone at a dose of 30 mg/kg slow IV once daily. On Day 21, the cardiac murmur increased to a grade 6/6 holosystolic murmur. Abdominal ultrasonography revealed no calcification of the kidneys or any other abdominal structures. Based on the expression of discoloured milk from the right half, the doe developed mastitis. A milk sample was collected aseptically for bacterial culture and sensitivity, which revealed *Staphylococcus* sp. growth. Clinical progression was poor, and on day 27, the doe was weaker, appeared to be more anxious, and was unable to stand, even with assistance. The animal was scheduled for a repeat echocardiogram to evaluate the progression of the cardiac lesions. Its heart rate was 120 BPM, with an irregular rhythm. Immediately after visualising the heart on echocardiography, an irregular beat pattern was observed, and the doe died while the electrocardiogram was being prepared. The animal did not respond to the initial resuscitation attempts, and re-animation procedures were discontinued due to concerns about long-term prognosis and animal welfare.

**Figure 3:**
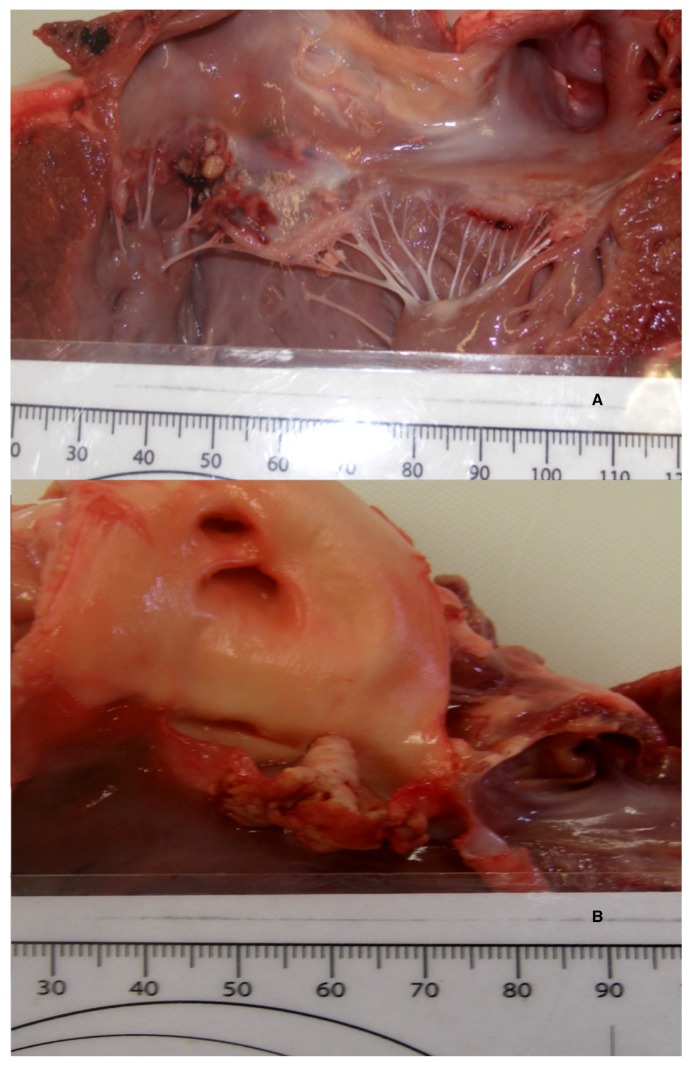
Vegetative fibrinonecrotic coalescing lesions at the mitral (**A**) and aortic valves (**B**).

**Figure 4:**
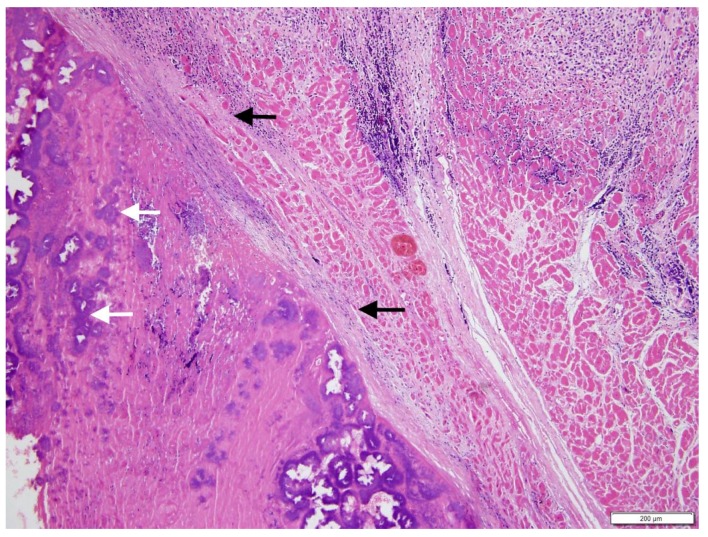
Effaced endocardium with attached fibrinonecrotic mass, containing bacterial colonies (white arrows), and separated from underlying myocardium by a zone of granulation tissue and fibrosis, which disrupts the cardiomyocytes (black arrows). H&E stain, magnification 10×.

A necropsy revealed a severe, fibrino-necrotic, vegetative mitral, aortic and tricuspid valve endocarditis (Figure 3A,B), with milder lesions on the tricuspid valve. A severe, unilateral, fibrino-suppurative, and haemorrhagic mastitis of the right udder, with severe, diffuse supra-mammary lymphadenitis, was also identified. There was moderate segmental enteritis and mild bronchopneumonia. Samples were collected for histopathology, and pericardial fluid and a fine needle aspirate, from the udder, were collected for cytology. Blood from the LV and secretions from the udder, as well as a section of the aortic valve, were collected for culture and sensitivity. The cytology for the pericardial fluid was consistent with acute haemorrhage. The cytological report for the secretion from the udder supported gross pathologic findings of a septic suppurative inflammation. On histological examination, the endocardium was multifocally effaced, with large attached masses of fibrin and necrotic debris, containing multiple bacterial colonies composed of gram-positive cocci forming groups. These areas were variably separated from underlying myocardium by a zone of granulation tissue and fibrosis, which extended into the myocardial interstitium separating the cardiomyocytes (Figure 4). Enzootic calcinosis was ruled out due to a lack of substantive gross and histopathologic findings. There was no colony growth for the vegetative lesion of the aortic valve or the blood from the LV. A culture of the secretions from the udder was performed following standard protocols for bacteriological culture. Briefly, isolates were placed on blood and MacConkey agar, colonies were subsequently Gram stained, and catalase and coagulase tests were performed. The culture results revealed catalase and coagulase positive *Staphylococcus* sp. growth, which was resistant to tetracycline, penicillin, and streptomycin.

## 3. Discussion

It has been documented that animals with conditions such as chronic active infections, including foot abscesses or rumenitis, can develop bacteraemia, which can be a predisposing factor for endocarditis [2]. Upon admission, the doe had a wound on the right teat with healthy granulation tissue. However, the character of the expressed milk was unremarkable. The doe remained recumbent, since admission, throughout the hospitalisation period. It is likely that the wound on the udder became infected due to the difficulty of maintaining proper hygiene in the affected area, leading to mastitis. The clinical diagnosis of mastitis was confirmed on necropsy both grossly and histologically and through culture of the secretions from the udder, which revealed *Staphylococcus* sp. growth. In this case, a blood culture yielded negative results. A post-mortem gram stain of the cardiac lesions showed the presence of multiple colonies of Gram-positive cocci forming groups. However, it was not possible to establish a clear link between the mastitis and endocarditis.

In the present case report, echocardiography and the distribution pattern of lesions suggested mitral “kissing vegetation”. Upon echocardiographic examination, the left coronary cusp of the aortic valve with a large vegetative growth, prolapsed into the left ventricle during diastole, contacting the septal leaflet of the mitral valve. Secondary involvement of the mitral valve is rare but well documented in primary aortic valve endocarditis in human species. This lesion results from a large aortic vegetation prolapsing into the left ventricle during diastole, making contact with the ventricular aspect of the anterior mitral leaflet, thus causing secondary infection [3,4,5]. In a study by Piper et al. [3], 19 out of 192 human patients with aortic valve infective endocarditis developed mitral valve lesions. They postulated that the majority of these patients had mitral valve involvement due to seeding of the infection from large aortic vegetations rather than contiguous spread, simultaneous infection or secondary damage due to “jet perforation” of the mitral valve. In addition, they concluded that patients with aortic valve endocarditis had an increased risk of developing secondary mitral infective endocarditis. In their study, vegetations were larger in size in patients with mitral valve involvement (>6 mm) [3]. Secondary involvement of the mitral valve, although uncommon, should be included in the list of possible complications of aortic infective endocarditis in animal species.

Aortic valve obstruction is an uncommon complication of infective endocarditis in humans and animals. Calcific degeneration of the aortic valve is the most common cause of acquired aortic stenosis in humans [6]. Calcification of cardiac valves, aortic endothelium and cardiac muscle with marked enlargement and vegetation-like structures in cardiac valves have been reported in goats, sheep and horses with enzootic calcinosis [7,8,9]. However, in our case there were few clinical signs, radiographic or ultrasonographic changes compatible with enzootic calcinosis. Furthermore, the animal had evidence of severe systemic inflammation and histopathology confirmed infective endocarditis. Distortion of the valvular tissue architecture due to endocarditis, has been shown to lead to acute acquired valvular obstruction in human beings [10,11,12]. Charney et al. described a patient with aortic stenosis due to infective endocarditis, which also had mitral involvement. Aortic valve stenosis was diagnosed based on echocardiogram and Doppler studies. Infective endocarditis was confirmed through histology of the affected valve after replacement [12]. In our case, there was a markedly decreased range of motion of the aortic valve during systole upon echocardiographic examination. In addition, there was an increased peak pressure gradient measured across the aortic valve adding evidence to the diagnosis of aortic valve obstruction. Acquired valvular stenosis due to infective endocarditis should also be taken into account when evaluating veterinary patients with infective endocarditis.

In the present case, a blood culture was taken, and it resulted in no growth of any organisms. In addition, a post-mortem culture of the vegetative lesion of the aortic valve and blood from the LV proved negative for bacterial growth. Negative culture endocarditis (NCE) can occur in up to 30% of human patients with infective endocarditis [13,14]. There are also reports of NCE in veterinary medicine [15]. It has been suggested that either the antibiotic treatment started prior to sampling, or the presence of fastidious bacteria, such as *Bartonella* sp. and *Coxiella* sp., may decrease the sensitivity of a blood culture for the diagnosis of infective endocarditis [13]. The goat described in this report was on an antibiotic treatment prior to blood and tissue cultures, which could have contributed to a negative result.

To the best of our knowledge, this is the first case report describing mitral kissing vegetation associated with infective endocarditis, involving multiple cardiac valves and with secondary aortic stenosis, due to a large vegetative lesion in a goat.

## 4. Conclusions

The secondary involvement of the mitral valve is a possible complication of aortic infective endocarditis in animal species. Infective endocarditis can cause acquired valvular obstruction. Negative blood and tissue cultures may occur in cases of bacterial endocarditis in goats.

## Figures and Tables

**Table 1:** Main haematological parameters on admission. Marked leukocytosis, neutrophilia, lymphocytosis, and mild monocytosis, with a mildly elevated total protein, were observed.

Parameter	Patients Values	Reference Intervals
WBC	31.50 × 10^9^/L	6 to 14 × 10^9^/L
Neutrophil	11.03 × 10^9^/L	1.2 to 7.2 × 10^9^/L
Lymphocytes	11.03 × 10^9^/L	2.0 to 9.0 × 10^9^/L
Monocytes	1.26 × 10^9^/L	0.0 to 0.5 × 10^9^/L
Plasma Proteins	83 G/L	62 to 79 G/L
Fibrinogen	1 G/L	1 to 5 G/L
Serum Calcium	2.12 mmol/L	2.3 to 2.9 mmol/L

## Supplementary Materials

The following are available online at http://www.mdpi.com/2306-7381/5/3/64/s1, Video S1: Left parasternal longitudinal view showing an aortic valve cusp with a vegetative growth prolapsing into the left ventricle during diastole and making contact with a mitral valve cusp (Mitral kissing vegetation).
